# Predicting Motor Outcomes in Stroke Patients Using Diffusion Spectrum MRI Microstructural Measures

**DOI:** 10.3389/fneur.2019.00072

**Published:** 2019-02-18

**Authors:** Kyler Hodgson, Ganesh Adluru, Lorie G. Richards, Jennifer J. Majersik, Greg Stoddard, Nagesh Adluru, Edward DiBella

**Affiliations:** ^1^Department of Biomedical Engineering, University of Utah, Salt Lake City, UT, United States; ^2^Department of Radiology and Imaging Sciences, Utah Center for Advanced Imaging Research, University of Utah, Salt Lake City, UT, United States; ^3^Department of Occupational and Recreational Therapies, University of Utah, Salt Lake City, UT, United States; ^4^Department of Neurology, University of Utah, Salt Lake City, UT, United States; ^5^Divison of Epidemiology, Department of Internal Medicine, University of Utah, Salt Lake City, UT, United States; ^6^Waisman Center, University of Wisconsin, Madison, WI, United States

**Keywords:** stroke, motor, NODDI, Fugl-Meyer, DSI, DTI, diffusion, MRI

## Abstract

Improved understanding of neuroimaging signal changes and their relation to patient outcomes after ischemic stroke is needed to improve ability to predict motor improvement and make therapy recommendations. The posterior limb of the internal capsule (PLIC) is a hub of afferent and efferent motor signaling and this work proposes new, image-based methods for prognosis based on interhemispheric differences in the PLIC. In this work, nine acute supratentorial ischemic stroke patients with motor impairment received a baseline, 203-direction diffusion brain MRI and a clinical assessment 3–12 days post-stroke and were compared to nine age-matched healthy controls. Asymmetries based on the mean and Kullback-Leibler divergence in the ipsilesional and contralesional PLIC were calculated for diffusion tensor imaging (DTI) and diffusion spectrum imaging (DSI) measures from the baseline MRI. Predictions of upper extremity Fugl-Meyer (FM) scores at 5-weeks follow-up from baseline measures of PLIC asymmetry in diffusion tensor imaging (DTI) and diffusion spectrum imaging (DSI) models were evaluated. For the stroke participants, the baseline asymmetry measures in the PLIC for the orientation dispersion index of the neurite orientation dispersion and density imaging (NODDI) model were highly correlated with upper extremity FM outcomes (*r*^2^ = 0.83). Use of DSI and the NODDI orientation dispersion index parameter shows promise of being more predictive of stroke recovery and to help better understand white matter changes in stroke, beyond DTI measures. The new finding that baseline interhemispheric differences in the PLIC calculated from the orientation dispersion index of the NODDI model are highly correlated with upper extremity functional outcomes may lead to improved image-based motor-outcome prediction after middle cerebral artery ischemic stroke.

## Introduction

After the acute phase of an ischemic stroke, the best course of treatment is often not clear. While measures such as baseline disability levels, age at stroke onset, infarct volume, and lesion location are used in predicting general stroke recovery outcomes, they are not used in predicting specific motor function outcomes such as upper extremity (UE) scores (1). Prognosis of post-stroke recovery is needed but remains imprecise. For example, clinicians do not know which patients can expect benefit from motor rehabilitation, nor whether to focus therapy efforts on restoring motor function or teaching compensatory strategies. Recommendations for therapy to improve UE function would benefit from more accurate methods during the first weeks post-stroke to predict potential for motor recovery. Many researchers have argued for the development of biomarkers to predict such recovery (2), with neuroimaging biomarkers being the most studied. The most common neuroimaging biomarkers studied for their ability to predict post-stroke UE recovery have been those measuring the integrity of corticospinal tract white matter (3–10).

Analysis of white matter integrity has most commonly been done with DTI measures such as fractional anisotropy (FA), mean diffusivity (MD), radial diffusivity, and axial diffusivity (AD). These measures have shown modest correlation with stroke outcomes (3, 4, 7–9). Furthermore, when paired with transcranial magnetic stimulation, DTI based measures have demonstrated clinical utility in stroke outcome prediction (6, 9). However, there remain many problems with clinical implementation of these approaches, from insufficient accuracy in prediction to the time-intensive nature of these assessments. Some of the inaccuracy of these methods stems from the limitations of DTI in discerning white matter integrity in regions of crossing fibers, trauma, and axonal remodeling (11, 12). Higher order diffusion methods with MRI may be able to provide greater prediction accuracy.

### Diffusion Spectrum Imaging (DSI) Models for Stroke Prognosis

MRI measures derived from diffusion spectrum imaging (DSI) using generalized fractional anisotropy (GFA) show promise to assess the integrity of the corticospinal tract (CST) and to predict motor function recovery. DSI may overcome the limitations of DTI and add useful detail regarding the extent of white matter degeneration in regions affected by stroke. If measures derived from DSI models provide better estimation of white matter integrity indicative of improved potential for motor function recovery, an image-only based prognosis system may be realized. At the very least, combination approaches of TMS and MRI for prognosis should be improved by DSI models with a fast acquisition scheme.

DSI generalizes DTI by acquiring more directions in q-space either through high angular resolution diffusion imaging (HARDI) shells, q-ball imaging or a cube on a Cartesian grid (13, 14). DSI is thought to better estimate areas of crossing or kissing fibers, demyelination (15), and axonal remodeling (16) missed in DTI. A popular measure estimated from DSI data and previously used to predict stroke outcome based on changes in white matter (5, 16, 17) is GFA. GFA is described as the standard deviation of diffusion directions in a voxel and is the DSI analog of the DTI derived parameter FA.

### Rationale Behind NODDI for Stroke

NODDI is a multi-compartment model that differs from prior models in that it estimates the intracellular and extracellular contributions to the signal in terms of neurite morphology. This means that the extracellular components of the signal are estimated in terms of the intracellular components, instead of separately estimating the compartments, or treating them as a single compartment. Such a multi-compartmental approach is thought to be useful in discerning areas of crossing fibers (15). NODDI parameters may also better estimate the integrity of white matter in the subacute phase of stroke when Wallerian degeneration and processes involving reactive astrocytes and microglia that lead to glial scarring are occurring (18).

### Aims

In this work, the efficacy of measures from multi-compartment and high-angular resolution models in predicting upper extremity motor function recovery in stroke patients was examined using data from a fast DSI acquisition scheme. (a) We first aimed to understand how ipsilesional and contralesional regions of the CST in participants with stroke deviate from controls (b) Next, we evaluated the ability to predict motor function outcomes at 5-week follow-up from measures of asymmetry in regions of the CST in a baseline scan. (c) Last, we investigated the association of baseline lesion size and CST lesion load with motor function outcomes.

## Materials and Methods

### Participant Selection and Clinical Assessments

This study was carried out in accordance with the recommendations of Department of Health and Human Services (DHHS) and Food and Drug Administration (FDA). The protocol was approved by the Institutional Review Board at the University of Utah (assurance number FWA00003745 - U of Utah). All subjects gave written informed consent in accordance with the Declaration of Helsinki.

Stroke patients were recruited through the University of Utah Stroke Center at the University of Utah hospital. Patient inclusion criteria were: (1) supratentorial, imaging confirmed, ischemic stroke; (2) sufficient upper extremity weakness with some voluntary movement ability; (3) baseline Fugl-Meyer (FM) Upper Extremity (UE) score ≤50 and FM Lower Extremity score ≤28; and (4) MRI scan taken >2 days and <2 weeks from stroke onset. FM assessments were performed by an occupational therapist within 2 weeks of the stroke date and 1 ± 3 days from the date of the baseline MRI scan. Follow-up Fugl-Meyer assessment was performed a mean ± standard deviation of 38 ± 9 days post-stroke for all subjects. This follow-up assessment we refer to as tp2, and, when referring to the Upper-Extremity FM scores, as FM UE tp2. Change in FM UE was defined as the difference between FM UE tp2 and FM UE baseline. See Appendix for summary of stroke patient data.

Non-stroke participants of similar age to stroke participants were recruited by the Department of Radiology and Imaging Sciences Research Staff. Control participants had no known history of neurological disorders.

All patients received usual neurologic and rehabilitation care and either sildenafil, memantine, or a placebo after undergoing a baseline MRI scan. Data were blinded to interventional information for this analysis since the intent was to determine whether a baseline scan has predictive value of eventual motor recovery, regardless of treatment.

### Data Acquisition

Fully sampled DSI data were acquired with equal spacing in q-space on a cartesian grid in 203 directions with a maximum b-value of 4,000 s·mm^−2^ for the 9 stroke participants and 9 non-stroke participants on a Siemens 3T Verio scanner using a 32 channel head coil 3–12 days post-stroke (19). A second scan was acquired 38 ± 9 days post-stroke for 8 participants. For all scans, a simultaneous multi-slice blipped controlled aliasing sequence (20) with a slice acceleration factor of three was employed. The scan parameters were *TR* = 3.7 s, *TE* = 114.2 ms, number of slices = 51, slice thickness of 2.1 mm, FOV 250 mm^2^, voxel dimensions 1.9 × 1.9 × 2.1 mm^3^, and a total data acquisition time of 12–13 min.

### Data Processing

First, skull stripping was performed on the reconstructed images using the brain extraction tool in (21). Noise was removed by identifying the noise-only principal components for local neighborhoods of voxels, the bulk of which are described by the universal Marchenko-Pastur distribution (22). Correction for noise-induced bias resulting from high diffusion weighting was done by transforming the magnitude of the signals to Gaussian signals according to (23, 24). Gibb's ringing was corrected by implementing software (25) that re-interpolated the image based on local, subvoxel-shifts to sample the ringing pattern at the zero-crossings of an oscillating sinc-function (26). Last, magnetic field inhomogeneities and eddy current induced distortion were corrected using the eddy_openmp command (27). These signal processing steps help remove many of the distortions and biases, enabling accurate estimation of the microstructural features. MATLAB 2017a was used to wrap all the processing steps to be executable from a single script.

**Figure 1:**
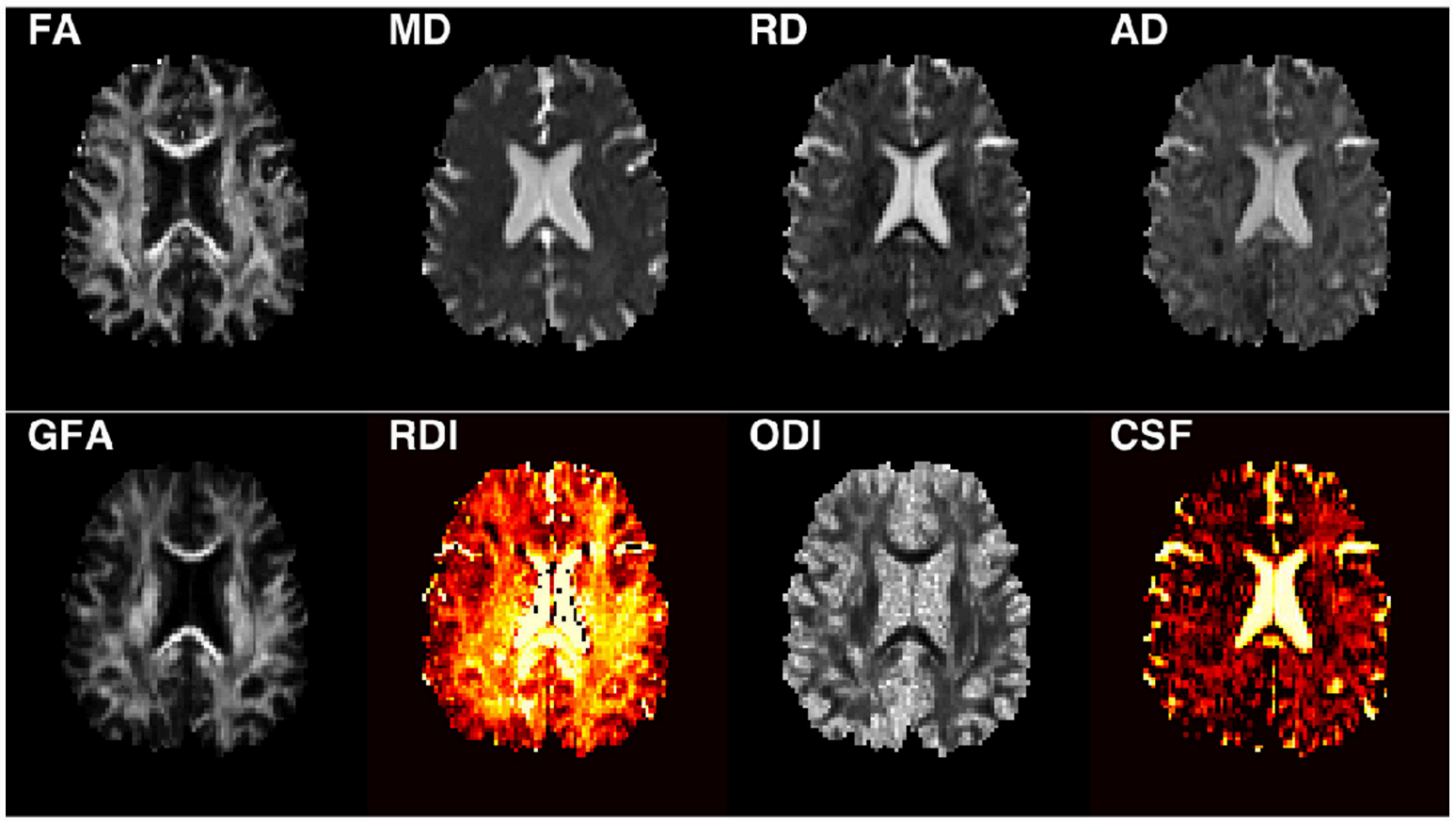
Example of a stroke subject image for each parameter investigated.

Following the processing steps, the diffusion tensor elements were estimated using non-linear optimization with positive definiteness constraints on the tensors using CAMINO (28) from which FA, MD, AD, and RD maps were calculated (Figure 1). The orientation distribution functions were estimated using DIPY (17, 29) for calculating GFA. The NODDI model was fit to the data using the NODDI MATLAB toolbox with default parameters (15, 30).

### Corticospinal Tract Label Estimation

Labels for white matter structures in the brain were estimated for all non-stroke and stroke scans, by warping the FA maps to fit the Johns Hopkins University FA 2.0 mm atlas (31, 32) using Advanced Normalization Tools (32, 33). Individual labels for the corona radiata, posterior limb of the internal capsule (PLIC), and cerebral peduncle, were identified and used in subsequent analysis.

## Analysis

### Deviation of Stroke Corticospinal Tract From Control

The mean values in the cerebral peduncle, PLIC, and corona radiata for each DTI and DSI parameter were calculated for all participants. A paired two-tailed *t*-test was performed in the cerebral peduncle, PLIC and corona radiata to determine whether significant hemispheric differences exist in controls in any region of the CST. In participants with stroke, a paired two-tailed *t*-test was performed for ipsilesional vs. contralesional segments of the CST. Last, significant differences between ipsilesional regions of the CST in participants with stroke and the CST in controls were investigated through two-sample two-tailed heteroscedastic *t*-test for each imaging parameter. *P*-values were adjusted for multiple comparisons using Holm's multiplicity adjustment, which controls the type I error without the need to first test the global hypothesis with ANOVA.

### Calculation of Microstructural Asymmetries in Baseline Stroke Corticospinal Tract

Because most prior studies report the PLIC to be the most important region of the CST in image-based motor function prognosis, the next steps of the analysis assessing the potential for DSI and DTI image-based motor function prognosis were focused on the PLIC.

Two different approaches were used to calculate the asymmetries for the PLIC for each DTI and DSI parameter map in each participant: **(1)** the interhemispheric mean difference, referred to as ΔPLIC_Mean_ and defined as,

(1) $$\begin{array}{r} \Delta PLIC_{Mean}\left(C,I\right)=\;\bar{C}-\bar{I}, \end{array}$$

where $\bar{I}$ is the mean value in the ipsilesional PLIC, and $\bar{C}$ is the mean value in the contralesional PLIC; and **(2)** the Kullback-Leibler Divergence (KLD), referred to as ΔPLIC_KLD_ and defined as,

(2) $$\begin{array}{r} \Delta PLIC_{KLD}\left(C,I\right)=\;\frac{1}{2}\left[\overset{n}{\underset{i=1}{\sum }}\left(C\left(i\right)\ln\left(\frac{C\left(i\right)}{I\left(i\right)}\right)\right)\right], \end{array}$$

where *I* is the distribution of values in the ipsilesional PLIC, and *C* is the distribution in the contralesional PLIC. ΔPLIC_KLD_ was calculated in R version 3.3.2 using 10 bins with the command KL.shrink from the *entropy* library (34). The KLD calculates an unbounded (0-Inf) logarithmic difference that provides a more complete estimate of the divergence of distribution *C* from distribution *I* than only a comparison of means.

It was hypothesized that a greater divergence in the baseline ipsilesional and contralesional PLIC distributions would correlate with worse functional outcomes at tp2. To test this hypothesis, the correlations of ΔPLIC_Mean_ and the log transform of ΔPLIC_KLD_ with FM UE tp2 and change in FM UE were evaluated using a combination approach. To push the limits of the small sample size, six points were selected as a training set, and three points left out for every possible combination of the data resulting in 84 repetitions. A linear regression line was then fit to each training set. The line of best fit was calculated by finding the normalized root mean square error for the test cases that produced the mean normalized root mean square error. The final normalized root mean square error reported in the results was computed for the line of best fit with all 9 data points. The optimism adjusted coefficient of determination (*r*^2^) was calculated by subtracting the difference of the *r*^2^ for each training set from the *r*^2^ of the full data set. The mean value of these differences was then subtracted from the *r*^2^ of the full data set and is the *r*^2^ reported.

### Stroke ROI and Lesion Load of Corticospinal Tract

**Figure 2:**
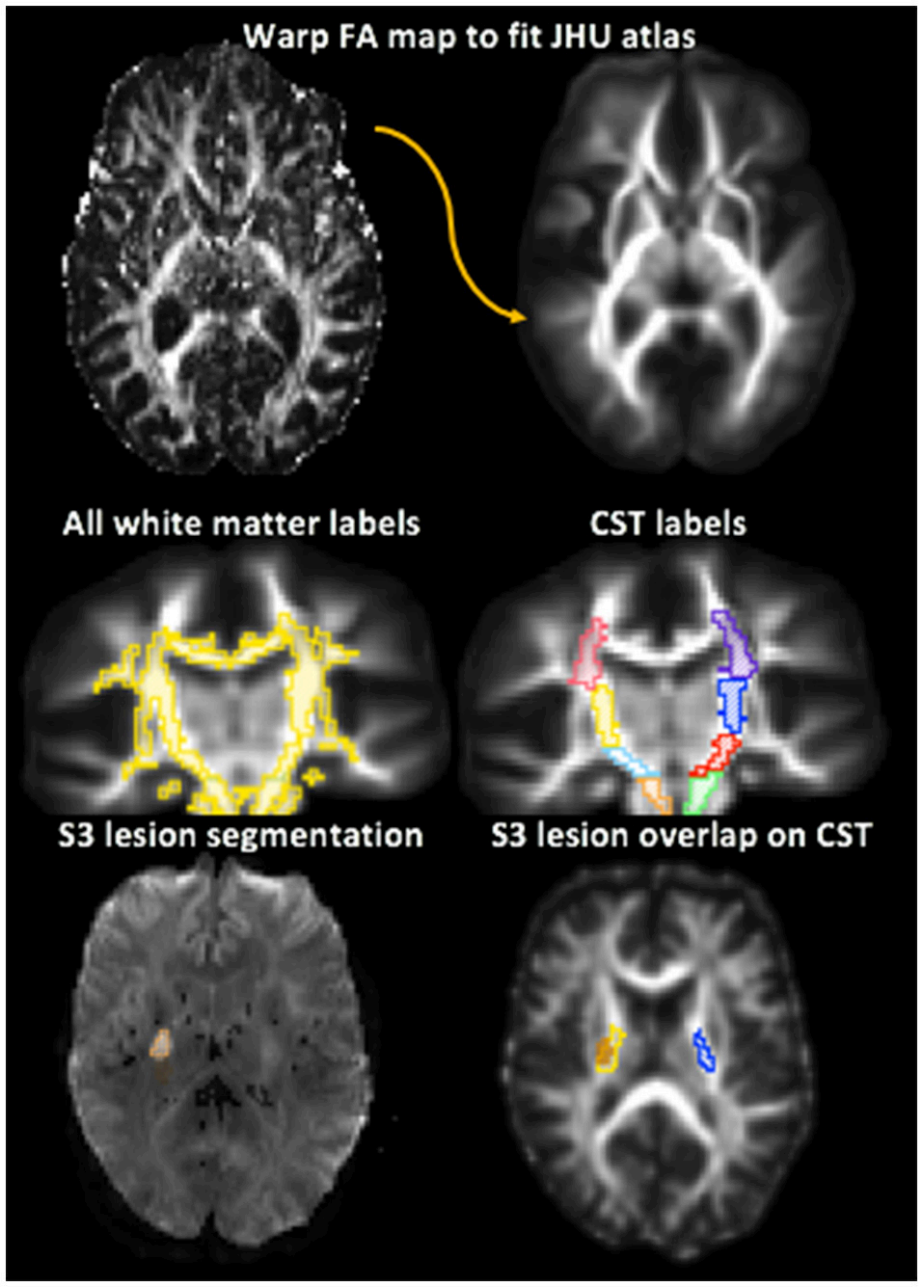
Depiction of white matter label transformation and segmentation of ipsilesional and contralesional stroke region of interest (ROI). Top: The Fractional Anisotropy (FA) map for each subject was warped to the Johns Hopkins University FA 2.0 mm Atlas. Middle left: The white matter labels were then transformed from the JHU atlas to the warped FA map. Middle right: Corticospinal tract (CST) regions were identified from the white matter labels. The PLIC is in yellow and blue. Bottom left: Stroke lesion ROI manually segmented. Bottom right: lesion load of the PLIC portion of the CST for stroke participant 3 (S3) shown by the overlap of the lesion ROI on the white matter labels. Lesion is shown in orange, right PLIC in yellow, and left PLIC in blue.

For each data set, the stroke region(s) of interest (ROIs) were confirmed from the radiologist report and the stroke ROIs manually drawn on DWI trace-weighted images in Seg3D (SCI) (Figure 2). The correlation of the lesion volume with baseline FM UE, FM UE tp2, and change in FM UE outcomes was then calculated. To calculate the baseline lesion load of the CST, the percent overlap of the stroke ROI with the Johns Hopkins University FA 2.0 mm atlas white matter labels for the corona radiata, PLIC, and cerebral peduncle was found. To evaluate the extent to which measures of asymmetry in the PLIC were affected by stroke location and the potential of baseline CST lesion load in motor outcome prediction, the correlation of the lesion load of each segment of the ipsilesional CST with baseline FM UE, FM UE tp2, and change in FM UE outcomes was calculated.

## Results

### Subjects

**Figure 3:**
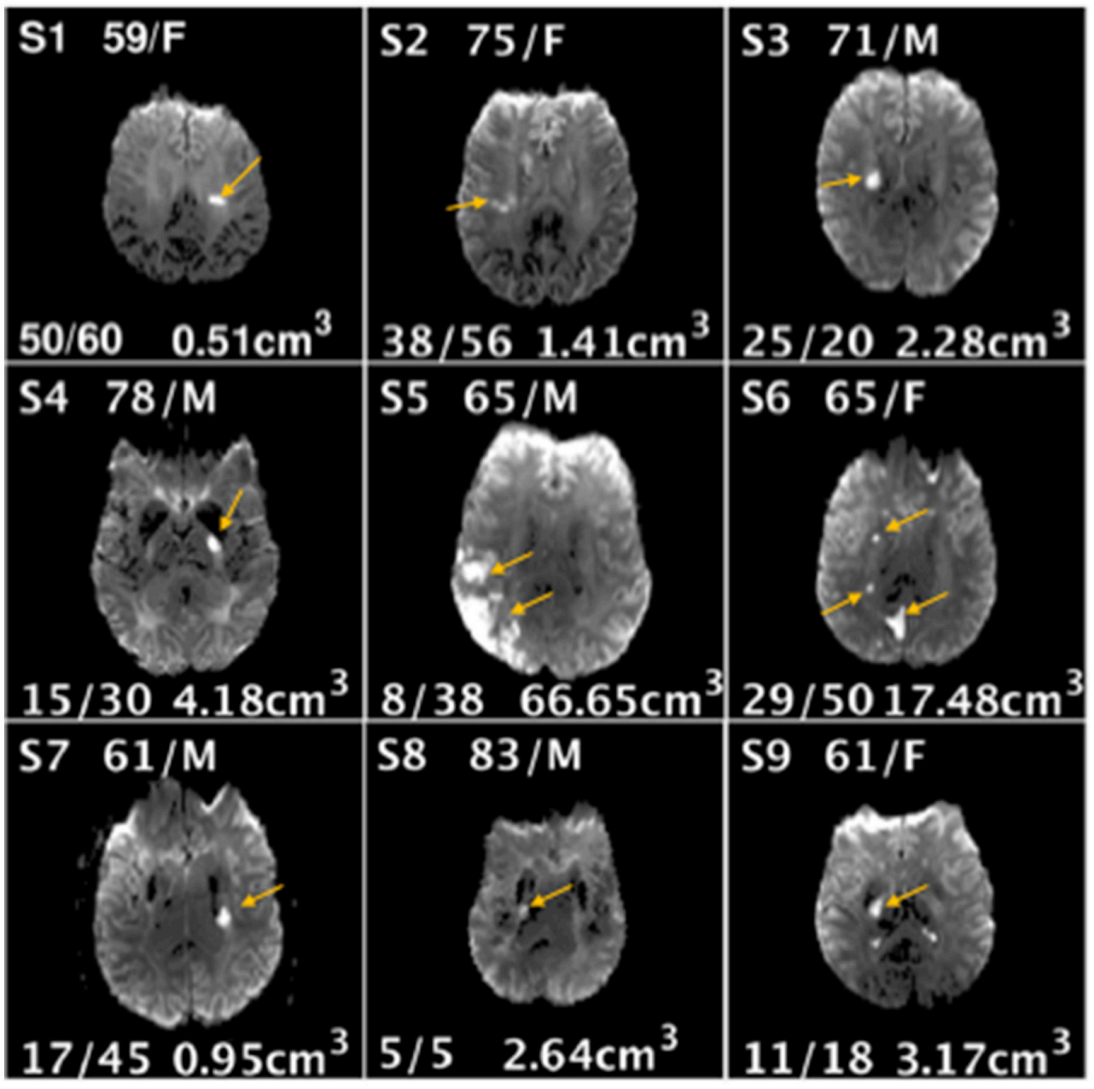
Demographic information age/gender (top center), Fugl-Meyer Upper Extremity scores at baseline and follow-up (bottom left), lesion volume (bottom right), and location (image shown) for each stroke subject (S1-S9).

The 9 participants with stroke were 69 ± 8.5 years in age and 44% were female. The 9 non-stroke participants were 67 ± 3.2 years in age and 11% were female. Strokes were of small-to-moderate size: volume of 12.04 ± 21.26 cc^3^ (range 0.51–66.65). Clinically, participants with stroke had an initial mean FM UE score of 22 ± 14.9 (range 5–50) and improved by tp2 to a mean FM UE score of 35.8 ± 18.8 (range 5–60). Seven of the nine patients improved with one showing no change and one worsening (Figure 3).

### Deviation of Stroke PLIC From Control

Using Holm's adjusted *p*-values, the left and right regions of the CST were not significantly different in any control participant. The ipsilesional PLIC was significantly different from the contralesional PLIC in stroke participants in ODI[tstat(DoF), *p*-value: *t*_(8)_ = 3.24, *p* = 0.0118], GFA [*t*_(8)_ = −3.52, *p* = 0.0077), FA [*t*_(8)_ = −3.59, *p* = 0.0071], and AD[t_(8)_ = −3.40, *p* = 0.0094]. Only FA showed a significant difference between hemispheres in the corona radiata *t*_(8)_ = −3.25, *p* = 0.0361. The contralesional segments of the stroke CST were not significantly from controls in the cerebral peduncle, PLIC, or corona radiata.

### Relation of Asymmetries in Baseline Stroke Corticospinal Tract to Functional Outcome

**Figure 4:**
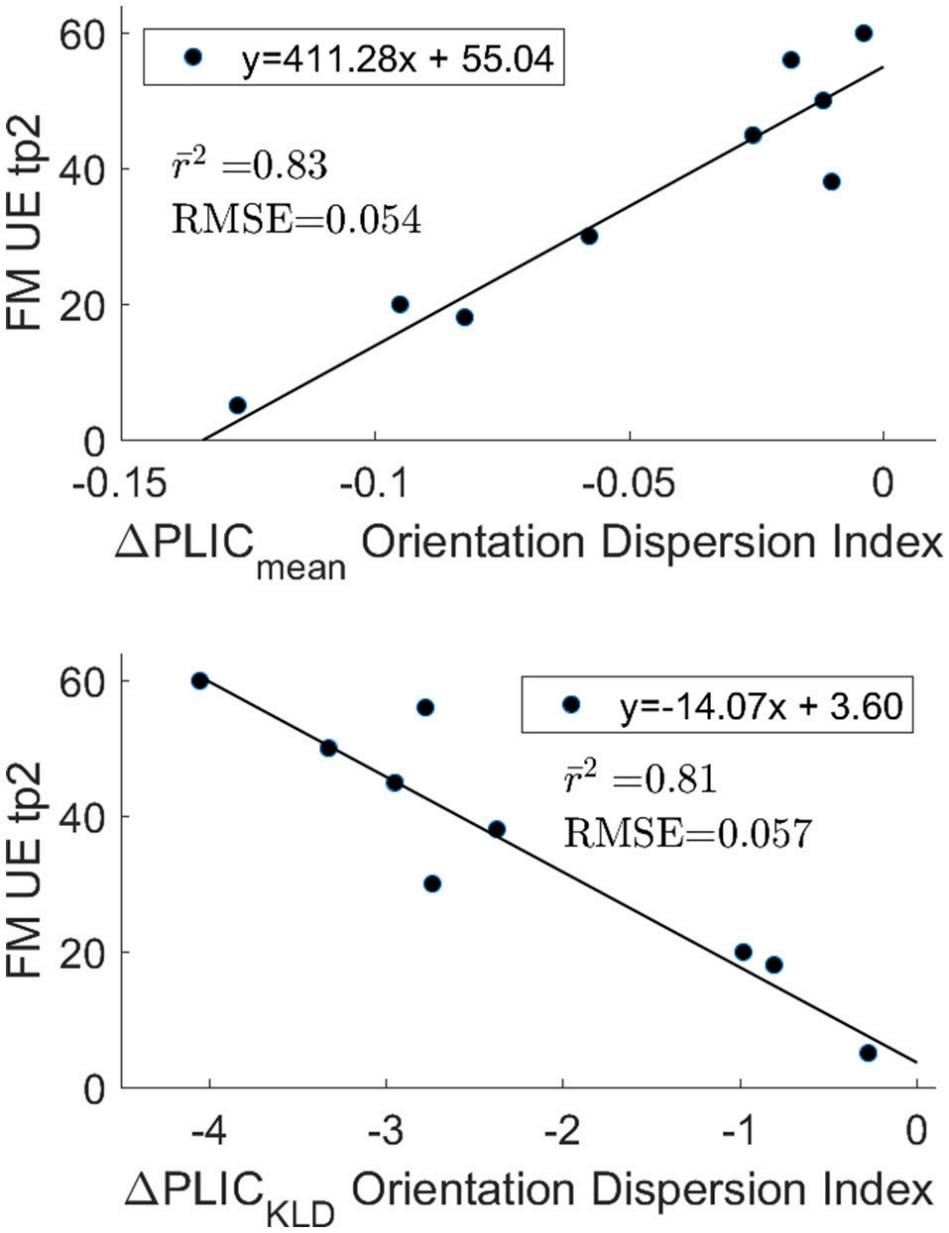
Optimism adjusted r2 after 84 permutations of n choose 6. The ΔPLIC_mean_ (top) and ΔPLIC_KLD_ (bottom) are highly correlated with functional outcomes and are in agreement with one another. The mean RMSE for the test data sets is also reported. The strong correlation observed between outcomes and the differences in ODI between hemispheres in the PLIC does not appear to be driven by extreme values for FM UE tp2 or ODI.

The optimism adjusted coefficient of determination after validation through combination testing showed encouraging results. The highest correlation for the mean difference with FM UE tp2 was ODI ΔPLIC_Mean_ (*r*^2^ = 0.83, rmse = 0.054, Figure 4). GFA ΔPLIC_Mean_ (*r*^2^ = 0.57, rmse = 0.099) also showed notable correlation with FM UE tp2. For the KLD measures, GFA ΔPLIC_KLD_ (*r*^2^ = 0.57, rmse = 0.097), RDI ΔPLIC_KLD_ (*r*^2^ = 0.70, rmse = 0.077), and ODI ΔPLIC_KLD_ (*r*^2^ = 0.81, rmse = 0.057) all had high correlation. The highest correlation with change in FM UE was for ODI ΔPLIC_Mean_ (*r*^2^ = 0.49, rmse = 0.175). The coefficient of determination of all other imaging measures with functional outcomes was below 0.5.

### Stroke Lesion Size and Lesion Load of CST

Baseline stroke lesion volume was not significantly correlated with baseline FM UE (*r*^2^ = 0.17 *p* = 0.28), FM UE tp2 (*r*^2^ = −0.01, *p* = 0.98), or change in FM UE (*r*^2^ = 0.24, *p* = 0.18). Lesion load was defined as the percent of the region of interest overlapped by the stroke lesion. The lesion load of the whole CST was not significantly correlated with baseline UE (*r*^2^ = 0.03, *p* = 0.63), UE tp2 (*r*^2^ = 0.28, *p* = 0.14), or change in FM UE (*r*^2^ = 0.37, *p* = 0.08). The lesion loads of the corona radiata, PLIC, and cerebral peduncle were also not significantly correlated with baseline FM UE, FM UE tp2, or change in FM UE. The best correlation of lesion load with FM UE tp2 was for the PLIC (*r*^2^ = 0.35, *p* = 0.09).

## Discussion

Our work shows potential additional utility of DSI based models in human motor stroke analysis compared to previously reported methods of image-based stroke prognosis. DSI-based analysis showed that baseline measures of PLIC hemispheric asymmetry calculated from the orientation dispersion index (ODI) parameter of the NODDI model are highly correlated with UE functional outcome. This is the first study to extensively study NODDI parameters in stroke UE outcome prediction and the results are encouraging. One reason we observed an improved outcome prediction based on ODI may be that the ODI parameter detects changes in the ipsilesional PLIC indicative of Wallerian degeneration missed by traditional DTI measures and single compartment DSI models that are not capable of fully capturing these changes in the first weeks post-stroke.

### Imaging Wallerian Degeneration

**Figure 5:**
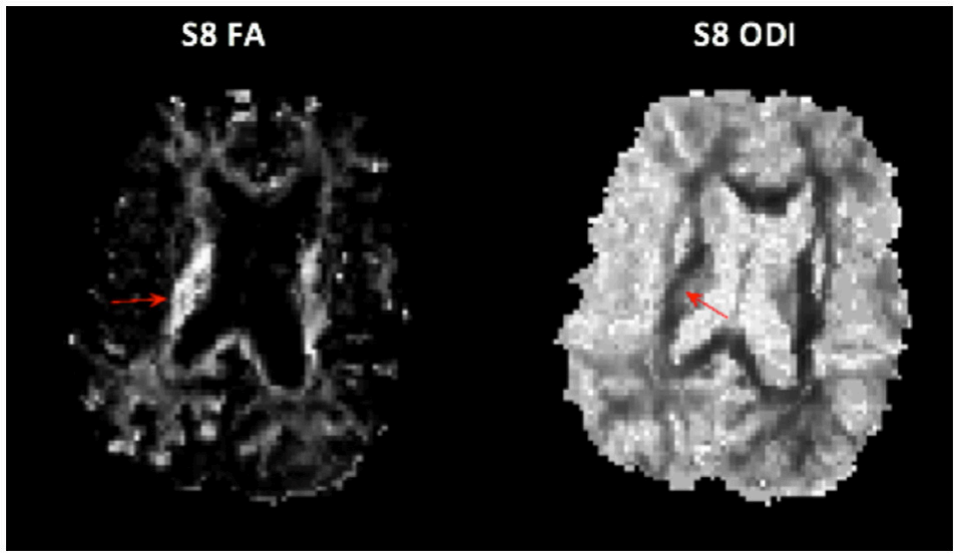
Unusual case of both elevated FA (**Left**) and elevated ODI (**Right**) in the PLIC in subject 8.

Although demyelination begins in the acute phase of stroke, many studies show that significant decreases in FA, thought to reflect Wallerian degeneration, are only detectable more than 25 days after stroke (3, 35, 36) Some studies report FA can detect degeneration <16 days post-stroke (37, 38) but the extent to which FA is reduced does not appear to be highly correlated with functional outcomes. The inability to reliably detect Wallerian degeneration at early time points with FA and other DTI parameters may be due to the response of glial cells involved in glial scarring. We did notice increased FA and ODI in some regions of the ipsilesional PLIC. These parameters are not normally both elevated simultaneously and may reflect ODI sensitivity to glial scarring (Figure 5).

ODI may better capture regions of glial scarring because the NODDI model is based on a multi-compartmental model that estimates intracellular and extracellular contributions to the observed signal in terms of neurite morphology (15, 39), instead of independently as in previous models (40, 41). Due to the dependency in the model, if the orientation dispersion of the intracellular compartment is increased, that is, myelinated axons have begun to degenerate, then the estimate of extra-cellular orientation dispersion will increase as well.

### Prediction of Stroke Outcomes From Baseline Asymmetries in the Posterior Limb of the Internal Capsule

#### DTI Estimated Asymmetries

Prior work based on DTI also found the differences in the PLIC region of the CST to be most predictive of outcomes (6, 7, 42, 43). A notable example is the PREP algorithm from Stinear et al., which utilizes a combination of FA and TMS to predict the potential upper extremity motor recovery after stroke (6). FA is used to calculate a normalized difference of mean values in the ipsilesional and contralesional PLIC called the asymmetry index, similar to ΔPLIC_Mean_ defined in this study (6). They reported that the asymmetry index correlated with recovery 12 weeks after stroke (*r* = −0.61, *p* < 0.001). In comparison, measures of asymmetry in the PLIC based on ODI, such as ΔPLIC_KLD_ and ΔPLIC_mean_ from this study, showed much higher correlation. The PREP algorithm was developed from a cohort of 40 participants, and a greater number of stroke participants would be needed to demonstrate that the image-only method describedhere is a suitable alternative to the PREP algorithm. In the meantime, ODI ΔPLIC_KLD_ and ODI ΔPLIC_mean_ could complement the FA based asymmetry index and improve the PREP algorithm. Last, DSI based measures of asymmetry in the PLIC could potentially improve upon DTI measures in predicting functional outcomes in response to motor rehabilitation or drug therapy from scans taken in the chronic phase of stroke (42–45).

### DSI Approaches to Stroke Outcome Prediction

Granziera et al. (5, 46), and Schulz et al. (47, 48) explored both interhemispheric and intrahemispheric differences in upper cortical regions, such as the primary motor and supplementary motor areas, shown in fMRI (49–51) to be active during recovery. In the Granziera et al. study, mean GFA in the connections between the motor cortex and subcortical structures in the contralesional hemisphere in individuals affected by stroke changed more than those in non-stroke controls. Using a multivariate regression analysis for their predictive model, baseline NIHSS scores, patient ages, and multiple mean GFA motor tract values at baseline accounted for 96 percent of the variance in NIHSS scores at 12 weeks. In this study, ODI ΔPLIC alone accounted for 83 percent of the variance in upper extremity outcomes at tp2. In (5), there was a relatively small cohort (12 stroke subjects) and therefore risked overfitting by using a multivariate approach. More study is needed to determine the performance of ODI ΔPLIC alone or included in a multivariate model.

Lin et al. expanded the GFA based motor tract analysis approach to include histogram derived measures: standard deviation, peak height, and skewness of GFA (16). The present study also benefited from the recognition that an analysis of the underlying distributional differences could better capture subtle variations in the microstructure. The current study differed from Lin et al. in that KLD was selected as a measure that captures the information gain between distributions. The KLD was near zero for nearly all control participants, meaning that the contralesional hemisphere nearly mirrors the ipsilesional hemisphere. In participants with stroke, it was found that a greater divergence of the ipsilesional PLIC and contralesional PLIC distributions for GFA, RDI, and ODI was correlated with poorer outcomes at a follow-up assessment. For ODI and GFA, ΔPLIC_KLD_ and ΔPLIC_mean_ were in agreement. On the other hand, KLD analysis for the RDI parameter showed better differentiation between and higher correlation with outcomes than the difference of mean values in the PLIC.

### Association of Baseline Lesion Size and Lesion Load of CST With Functional Outcomes

Similar to other groups, the correlation of the baseline ipsilesional CST lesion load with UE recovery was investigated (4, 7, 9, 45, 52–54). Most recently, Cassidy et al. found that the percent injury (lesion load) to the CST (cerebral peduncle and PLIC) after stroke, was significantly correlated with UE improvement after therapy (*r* = −0.41; *p* = 0.004; *n* = 47). Though no significant results for the entire CST lesion load were found in this study, the modest correlation with motor function outcomes observed agreed with previously reported studies (4, 9, 55). Breaking down the CST into individual segments yielded similar results, with the lesion load of the PLIC showing higher correlation with outcomes than the other segments, but still not reaching significance.

The PLIC is known to be one of the most integral brain regions to motor function. Simply knowing that a lesion is located in the PLIC would seem to be enough to predict motor function outcomes (4, 56). However, these results show that DSI based measures such as the NODDI ODI parameter, and to a lesser extent RDI and GFA, capture important information about the state of crucial areas of white matter that are not captured as well by simply estimating the percent of the structure overlapped by stroke lesion. Additional work shown in the Supplementary Material shows that correlation of the ODI values in the PLIC is driven more by values of the nonlesion areas of the PLIC than the lesion areas.

## Conclusions

The ODI parameter of the NODDI model was found to be the best measure of baseline asymmetry in the PLIC and predictive of upper extremity motor outcomes approximately 5 weeks after stroke. In ODI maps, mean differences in the PLIC and the KLD showed agreement. Overall, DSI based measures of PLIC asymmetry were more highly correlated with outcomes than DTI based measures. Lesion size and lesion load of the CST were not significantly correlated with outcomes. Future work will include a larger patient cohort and focus on analysis of longitudinal changes in areas critical to stroke outcomes captured through DSI parameters.

## Data Availability

Release of datasets would require additional IRB approval and might require re-consent of participants. Therefore, requests to access the datasets should be directed to Dr. Lorie Richards, lorie.richards@hsc.utah.edu and Dr. Jennifer Majersik, Jennifer.Majersik@hsc.utah.edu, who can apply to the IRB for approval.

## Ethics Statement

The University of Utah Institutional Review Board (IRB) approved the study protocol. All human participants provided written informed consent in accordance with the Utah IRB guidelines which are in accordance with the Declaration of Helsinki.

## Author Contributions

KH wrote the paper, processed the images, designed and performed the analysis, computed each of the models, and examined the relevant literature to find how the results fit in context of prior work. GA helped design the experiments, implemented the acquisition protocol, assembled some of the pre-processing techniques, and edited the manuscript. NA helped write the pre-processing code and was instrumental in choosing the k-fold analysis method and in writing some of the code to perform that analysis. Also performed editing of the methods and introduction sections. LR oversaw the Fugl-Meyer assessments and was instrumental in setting up the study and wrote some of the methods section and edited the manuscript. JM was instrumental in enrolling patients and setting up the study and performed editing throughout. GS contributed statistical expertise. ED conceived the research direction, assisted in design of the experiments, edited the manuscript, and was responsible for funding the imaging portions of the study.

### Conflict of Interest Statement

The authors declare that the research was conducted in the absence of any commercial or financial relationships that could be construed as a potential conflict of interest.

## Abbreviations

AD: axial diffusivity
CST: corticospinal tract
DSI: diffusion spectrum imaging
FA: fractional Anisotropy
FM: Fugl-Meyer
FM UE tp2: upper extremity Fugl-Meyer score at time point 2
GFA: generalized fractional anisotropy
MD: mean diffusivity
NODDI: neurite orientation density and dispersion imaging
PLIC: posterior limb of the internal capsule
RD: radial diffusivity
RDI: restricted diffusion index
UE: upper extremity
DoF: degrees of freedom.
