# Supplementary material for: Predicting Motor Outcomes in Stroke Patients Using Diffusion Spectrum MRI Microstructural Measures
Source: Front Neurol. 2019 Feb 18;10:72. doi: 10.3389/fneur.2019.00072 (PMC6387951; doi:10.3389/fneur.2019.00072)
Supplement: Supplementary file 1 [file Data_Sheet_1.PDF]

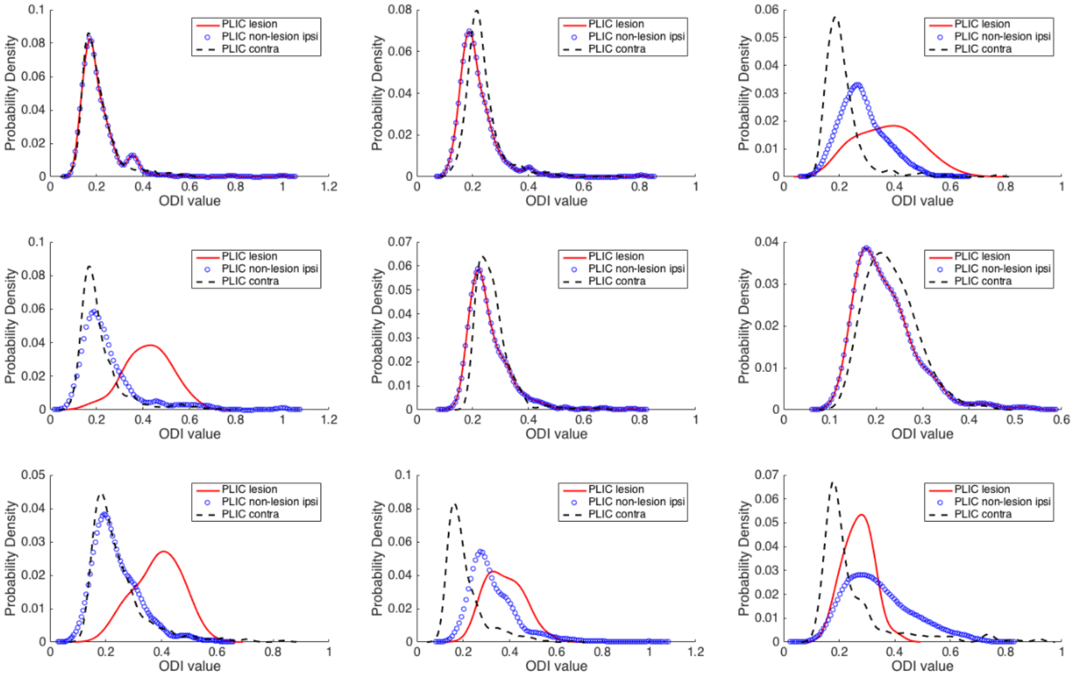

Supplementary Figure 1 – Distributions of the PLIC ipsilesional lesion area, the PLIC ipsilesional non-lesion area, and the contralesional PLIC. The total distributions of the ipsilesional PLIC are more closely aligned with the nonlesioned area. In general, the greater the difference between the ODI ipsilesional nonlesion PLIC and the contralesional ODI PLIC, the poorer the upper extremity outcomes.
