# Supplementary material for: Predicting Motor Outcomes in Stroke Patients Using Diffusion Spectrum MRI Microstructural Measures
Source: Front Neurol. 2019 Feb 18;10:72. doi: 10.3389/fneur.2019.00072 (PMC6387951; doi:10.3389/fneur.2019.00072)
Supplement: Supplementary file 2 [file Data_Sheet_2.PDF]

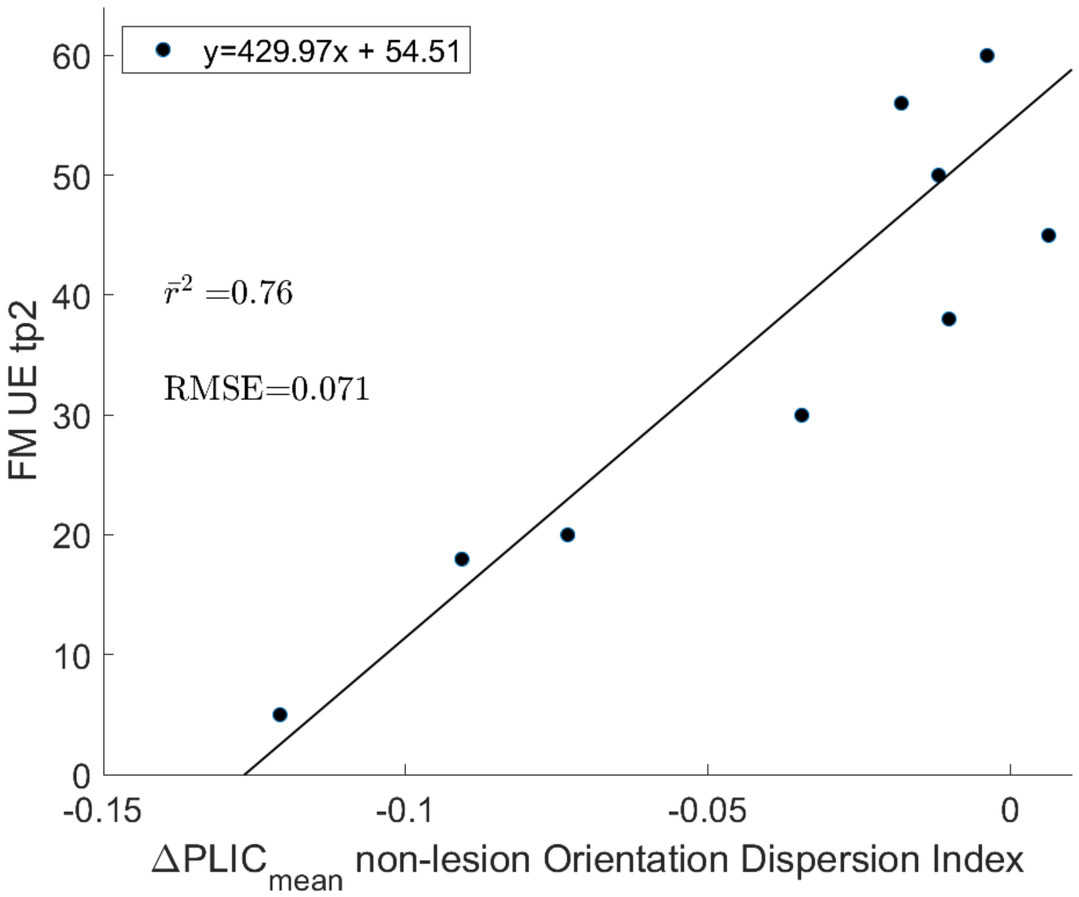

Supplementary Figure 2 – Mean difference of ipsilesional nonlesion PLIC ODI and the contralesional PLIC ODI. Masking the lesion out and analyzing only the nonlesion areas of the ipsilesional PLIC gives much better predictive value than the ODI value in the lesion alone. However, the optimism adjusted r^2 is slightly lower than the $\Delta PLIC_{mean}$ for the combined lesion and nonlesion areas of the ipsilesional PLIC. It is likely that some of the variance is explained by lesion load which is why including the lesion and nonlesion areas of the PLIC in the $\Delta PLIC_{mean}$ explained more of the observed variance than either alone.
